# The Matrix Approach to Patellar Instability

**DOI:** 10.7759/cureus.67703

**Published:** 2024-08-24

**Authors:** Tareq Almessabi, Dania W Al Rabaee, Ali H Ismaeil, Nabila Khan, Sattar Alshryda

**Affiliations:** 1 Trauma and Orthopaedics, Rashid Hospital, Dubai, ARE; 2 Orthopaedics and Trauma, Dubai Health, Dubai, ARE; 3 Trauma and Orthopaedics, Al Jalila Children's Speciality Hospital, Dubai, ARE; 4 Pediatric Orthopaedics and Trauma, Al Jalila Children's Speciality Hospital, Dubai, ARE

**Keywords:** matrix approach, dislocation, subluxation, patellofemoral instability, patella

## Abstract

Patellar instability is a challenging orthopedic condition affecting both pediatric and adult populations. The diagnosis and treatment of this condition present challenges for surgeons because of the multitude of classifications and treatment options available in the literature, leading to potential confusion in treatment strategies. Nonoperative treatments often prove ineffective, with reported recurrence rates nearing. Consequently, numerous surgical interventions have been developed in pursuit of improved outcomes. However, the results of these early interventions have not been universally successful, resulting in over 100 surgical interventions being recommended for patellofemoral instability, and none of which have achieved universal success. This hesitancy among surgeons to recommend surgery can leave patients inadequately treated. This article aims to share our matrix approach to patellar instability, developed over the past decade. By providing insights into the condition, we hope to stimulate interest among aspiring surgeons and facilitate a comprehensive understanding of the diagnosis and management of patellofemoral instability.

## Introduction

Patellofemoral dislocations represent a relatively common affliction in children and adults, with an estimated incidence of approximately 43 per 100,000 [1]. Patellar instability encompasses a broad spectrum of disorders, ranging from patellofemoral pain with no discernible clinical or radiological findings to a frank patellar dislocation, characterized by the patella residing outside the patellar groove. Intermediately, patellofemoral maltracking manifests not only in coronal misalignment but also in sagittal and axial axes.

Various elements affecting the stability of the patellofemoral joint include bony structure, ligament integrity, muscular strength, and instances of trauma [2]. Neglecting to account for these etiopathological factors in each patient with patellofemoral instability can compromise the efficacy of interventions. Moreover, numerous surgical approaches have been suggested, but none have garnered widespread success.

## Technical report

The matrix approach was developed to address this deficiency in clinical practice, and it does not deviate from other approaches. It relies on obtaining a comprehensive medical history, conducting a thorough physical examination, and ordering diagnostic tests, with particular emphasis on specific factors delineated by the matrix approach, including bony structure, ligamentous integrity, muscle strength, and the presence of traumatic elements (Table 1).

**Table 1:** Tabulated risk factors for patellar instability. Several bones, ligaments, and muscle abnormalities have been identified as risk factors for patellar instability. Trauma is another risk factor that can cause patellar instability directly or indirectly by affecting bones, ligaments, or muscles. mpFL: medial patellofemoral ligament; TTTG: tibial tubercle-trochlear groove

Bones	Ligaments	Muscles	Trauma
Femoral torsion	mPFL	Weakness	Accident
Tibial torsion	Generalized laxity	Hypotonia	Habitual (no accident)
Valgus knee alignment		Hypertonia	
Trochlear dysplasia			
Patellar dysplasia			
Patella alta			
High TTTG			

**Figure 1:**
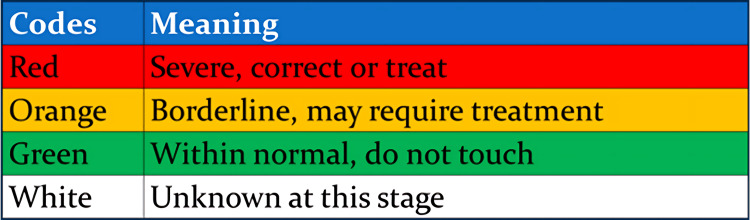
The matrix approach traffic light coding system to assess the severity of the risk factors. Red color denotes severity necessitating treatment, orange indicates borderline cases possibly requiring intervention, and green signifies normality where intervention is unnecessary. A white code is assigned when information is insufficient to determine whether a factor should be classified as green, orange, or red. Figures are provided by the authors.

Each of these factors is evaluated and categorized using a traffic light system: red denotes severity necessitating treatment, orange indicates borderline cases possibly requiring intervention, and green signifies normality where intervention is unnecessary. A white code is assigned when information is insufficient to determine whether a factor should be classified as green, orange, or red. Generally, a factor is classified as green if it falls within two standard deviations (SD) of the norm or average, as orange if it exceeds two SD but remains within three SD, and as severe if it surpasses three SD (Figure 1).

Bone structure

Eight bony irregularities have been recognized as potential risk factors for patellar instability. These include femoral torsion, tibial torsion, valgus knee alignment, trochlear dysplasia, patellar dysplasia, patella alta, and an elevated tibial tuberosity trochlear groove distance (TTTG).

Femoral and Tibial Torsion

**Figure 2:**
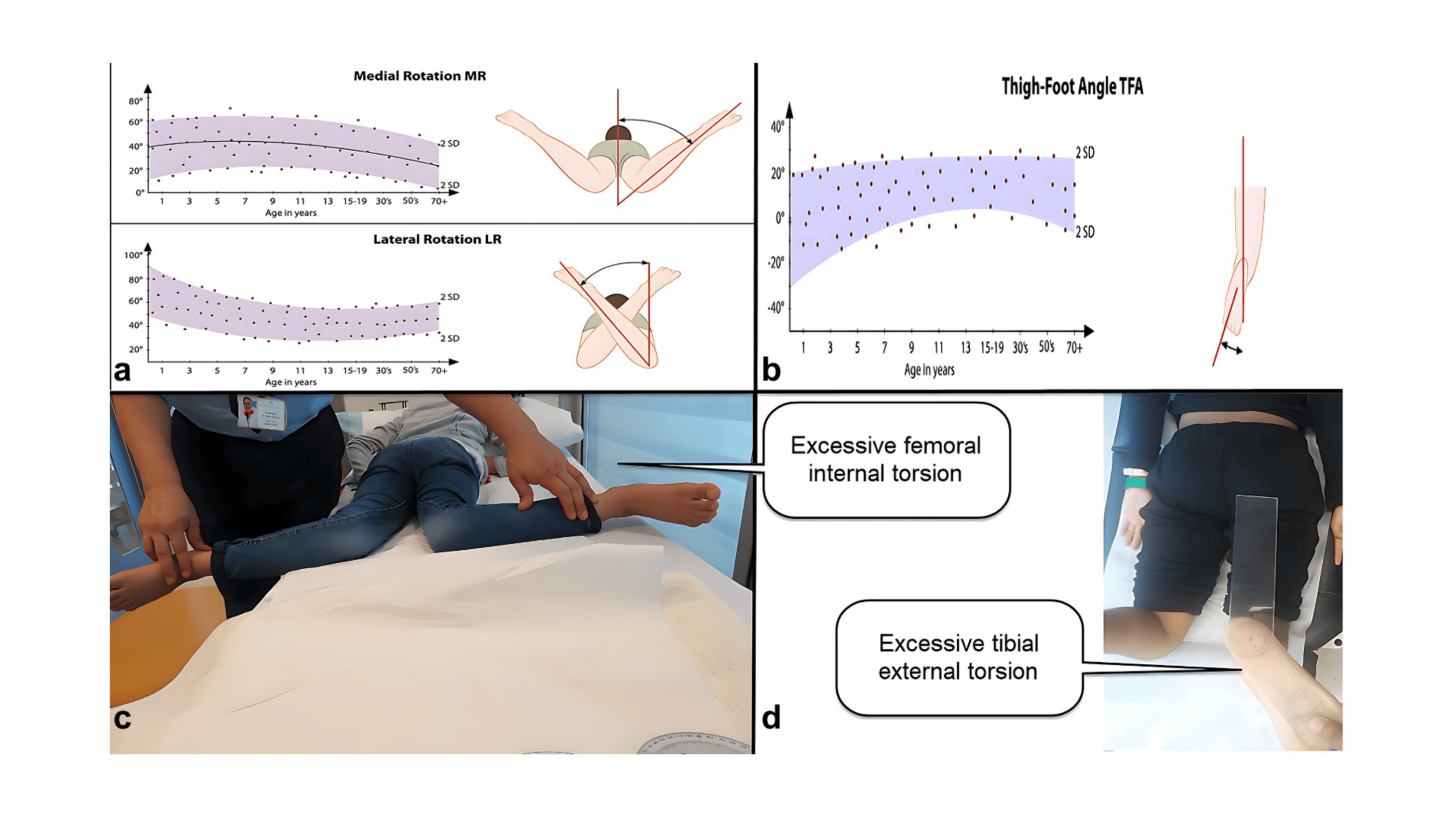
Femoral and tibial torsion are recognized risk factors for patellar instability. Figure 2a illustrates the typical range of femoral internal rotation, while Figure 2b depicts a child exhibiting excessive femoral internal rotation. Figure 2c displays the standard range for the foot-thigh angle, serving as an indicator of tibial external torsion, while Figure 2d presents a child with excessive tibial torsion. Figures are provided by the authors.

**Figure 3:**
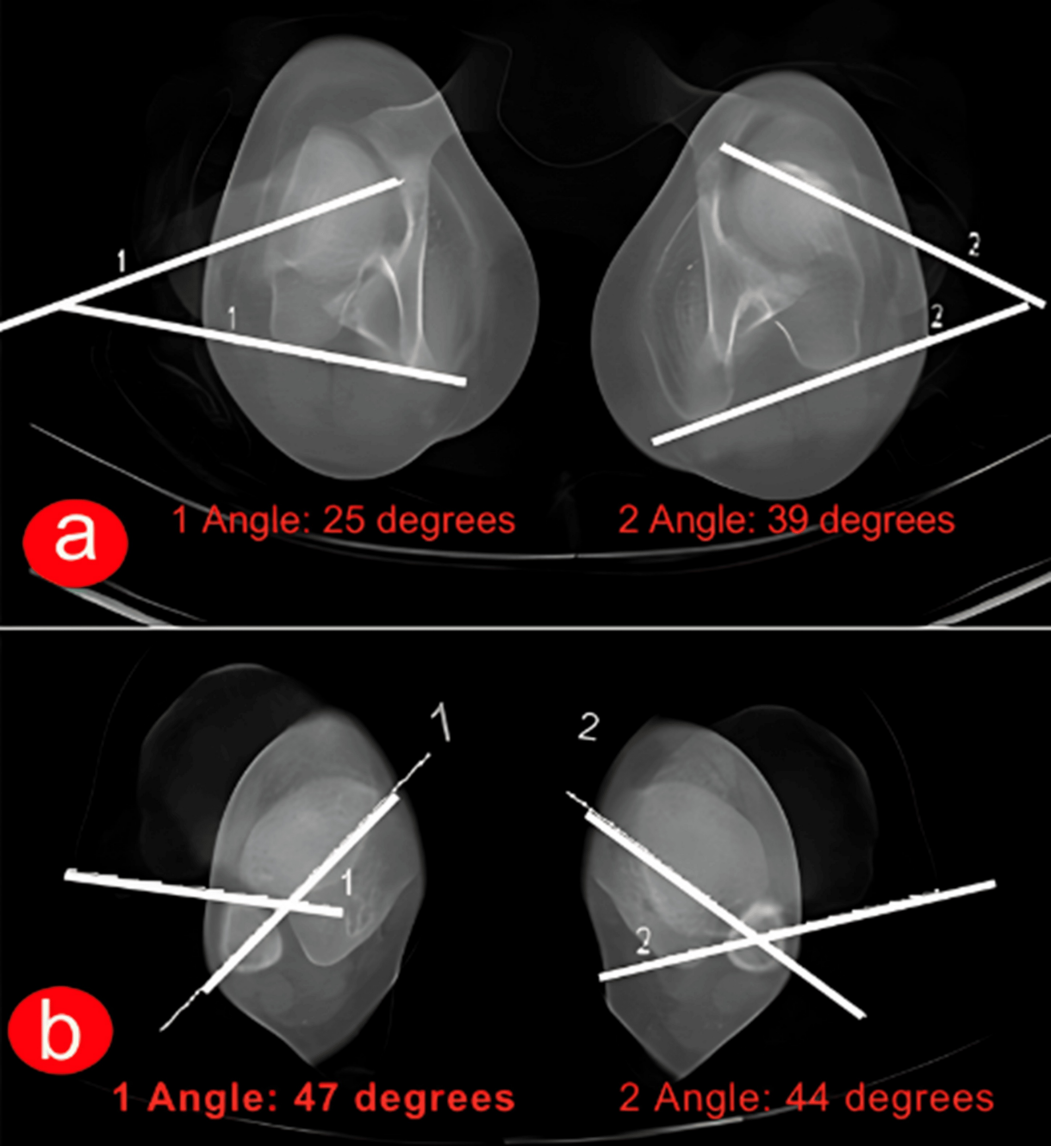
A CT scan of the lower limb’s rotation profile. A CT scan is employed to validate both femoral (as shown in Figure 3a) and tibial torsion (as depicted in Figure 3b). Figure 3a indicates a greater degree of femoral torsion on the left side (measuring 39°) compared to 25° on the right side. Conversely, the tibial torsion of both tibias appears to be nearly symmetrical. Figures are provided by the authors.

Femoral and tibial torsion are measured clinically and confirmed radiologically using CT scans (Figures 2-3, respectively) [3]. Values are compared against standard charts. Most femoral and tibial torsions tend to correct spontaneously during childhood. However, those that do not resolve naturally or necessitate immediate correction can be surgically addressed through derotational osteotomy [4].

Valgus Knee

**Figure 4:**
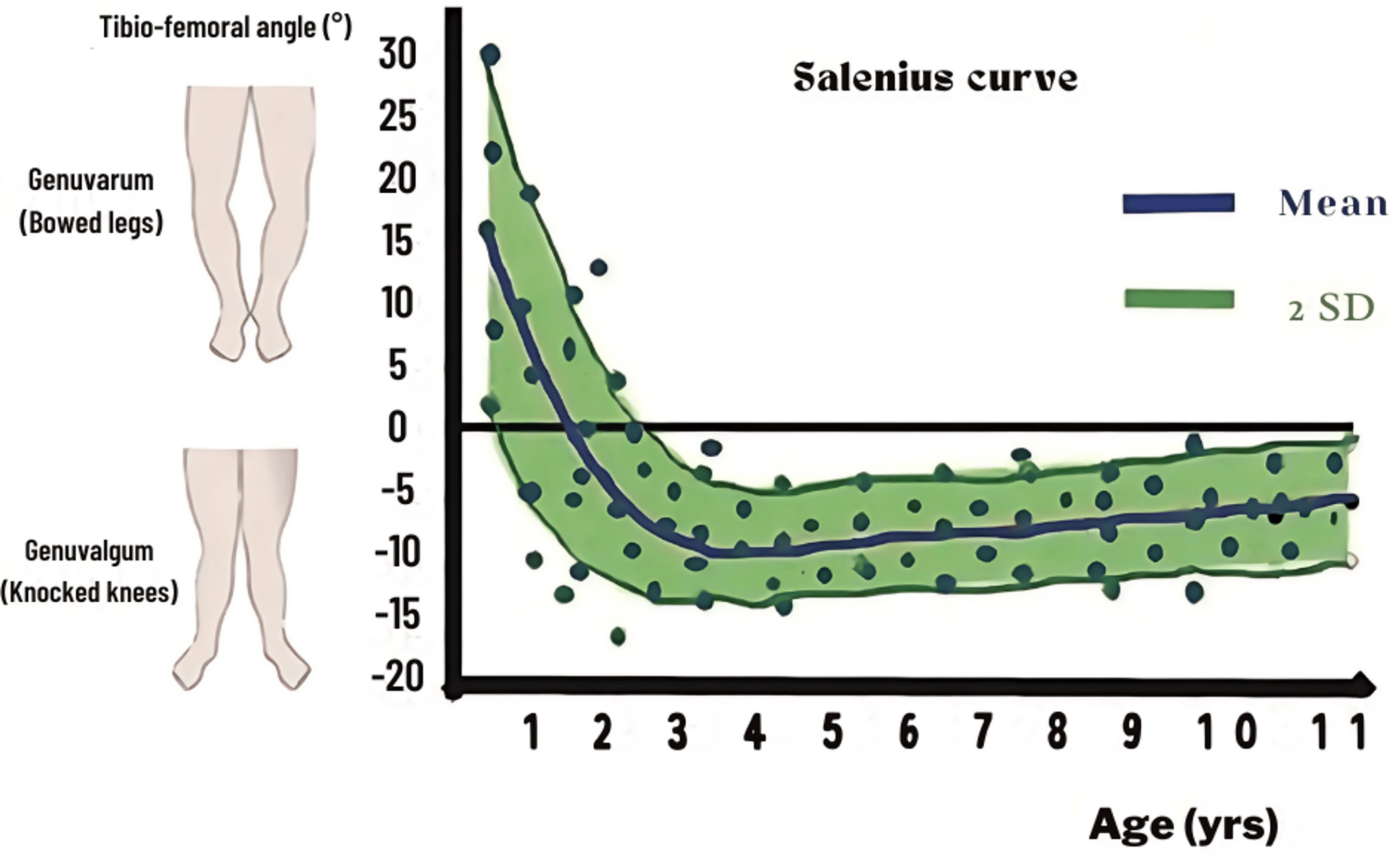
The Salenius curve. The Salenius curve demonstrates the alignment of the lower limbs, indicating whether there is a valgus or varus alignment based on the age of the patients. Figures are provided by the authors.

Valgus knee alignment can be assessed using three distinct methods: the angle between the femur and the tibia, the quadriceps angle (Q-angle), and the zone of the mechanical axis. The femur-tibia angle is typically evaluated via X-ray, utilizing the mid-diaphyseal line between the tibia and femur. Alternatively, a clinical assessment may be employed, with measurements falling within two SDs depicted as green on the Salenius curve (Figure 4 ), while deviations beyond this range are categorized as orange (within 3rd SD) or red when the measurement is beyond 3 SDs.

The Q angle is determined by drawing a line from the anterior superior iliac spine (ASIS) to the center of the patella, and another line from the center of the patella to the tibial tuberosity. In males, a normal Q angle is 10° or less, while in females it is 15° or less. A Q angle above 20° is classified as red, while values falling between these thresholds are categorized as orange. However, we argue that the Q angle may not be a reliable indicator of patellar instability as its value is influenced by the patella's position. If the patella is laterally dislocated, the Q angle measurement may inaccurately appear lower than expected.

**Figure 5:**
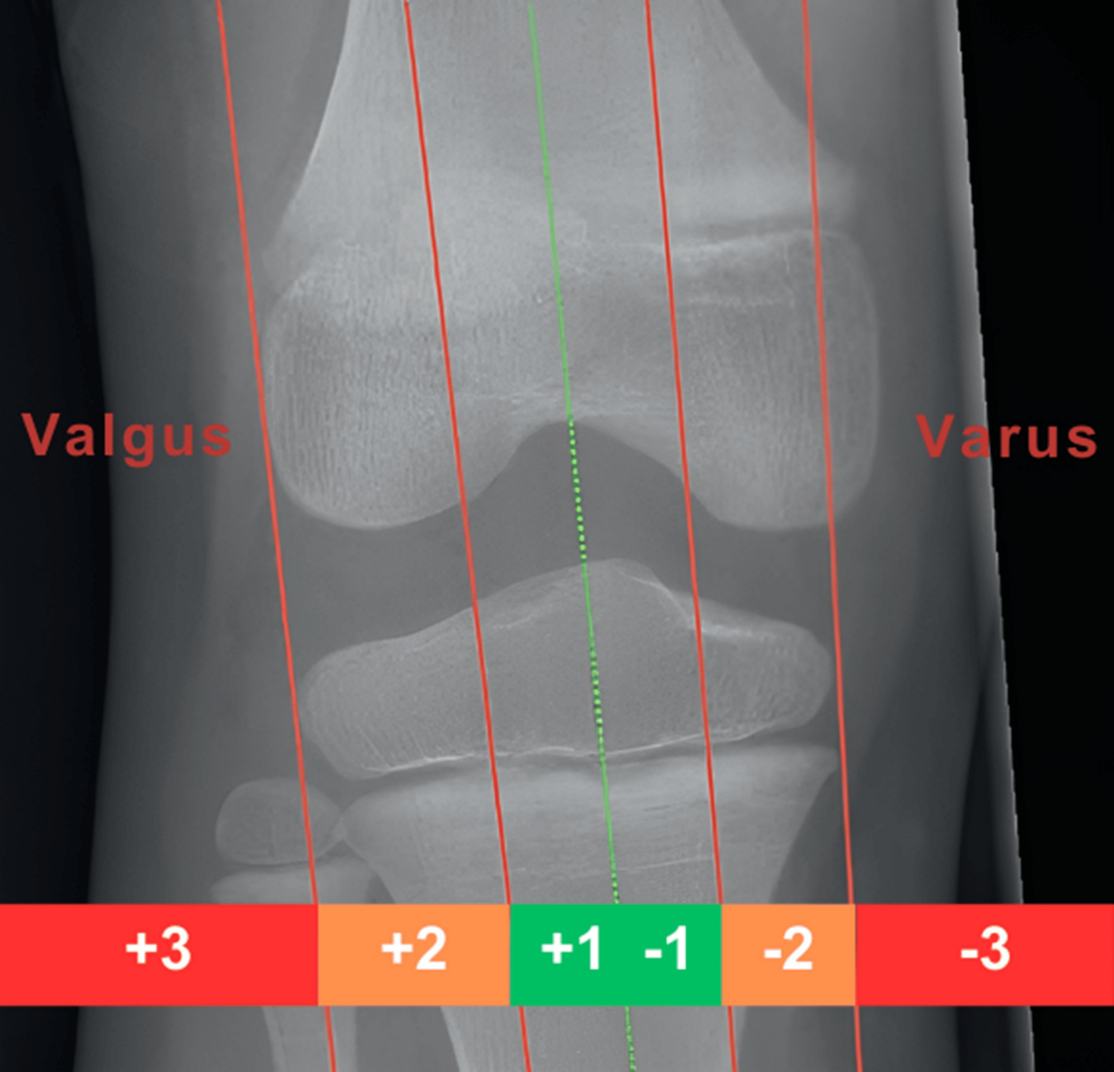
Zones of mechanical axes. Zone 1 encompasses the area around the knee center and femoral notch. Zone 2 covers the femoral condyle, while Zone 3 extends beyond the femoral condyle. Figures are provided by the authors.

The final method, and our favorite, is the zones of the mechanical axis (Figure 5). Three distinct zones are determined based on the intersection of the lower limb mechanical axis with the tibia plateau.

Zone 1 encompasses the area around the knee center and the femoral notch. Zone 2 covers the femoral condyle, while Zone 3 extends beyond the femoral condyle. A mechanical axis within Zone 1 is regarded as green or normal. However, if it falls within Zone 2, it is categorized as orange or borderline, and any alignment outside of the knee, in Zone 3, is considered severe and necessitates correction.

Valgus knee alignment can be addressed through guided methods while the patient is still in the growing phase or through corrective osteotomy.

Trochlear and Patellar Dysplasia

Trochlear dysplasia is characterized by a shallow, flattened trochlear groove, where the patella articulates. It is strongly associated with recurrent patellar instability, with approximately 85% of patients experiencing recurrent instability also presenting with trochlear dysplasia [5,6]. Beyond its role in patellar instability, trochlear dysplasia may predispose the patellofemoral joint to osteoarthritis. Biomechanical studies have demonstrated that the abnormal anatomy associated with trochlear dysplasia leads to increased contact pressure and aberrant patellofemoral kinematics [7].

Various radiological parameters have been proposed to assess trochlear dysplasia, among which is the Sulcus angle. This angle should ideally be less than 144°; values exceeding this threshold are considered abnormal. De Joure's classification system categorizes trochlear dysplasia into types A, B, C, and D based on lateral knee radiographs and cross-sectional cuts through the trochlea [8].

In type A dysplasia, a crossing sign is observed on lateral radiographs, and the trochlear groove appears symmetric but shallower than normal, with a sulcus angle greater than 145° on axial images. Type B dysplasia also presents with a crossing sign and a supratrochlear spur on lateral radiographs, accompanied by a flat trochlea on axial images. Type C dysplasia is characterized by a crossing sign and double contour on lateral radiographs, with lateral facet convexity and medial facet hypoplasia on axial images. Lastly, type D dysplasia exhibits a crossing sign, supratrochlear spur, and double contour on lateral radiographs, along with a "cliff" on axial images because of asymmetry of the lateral and medial femoral trochlear facets.

**Figure 6:**
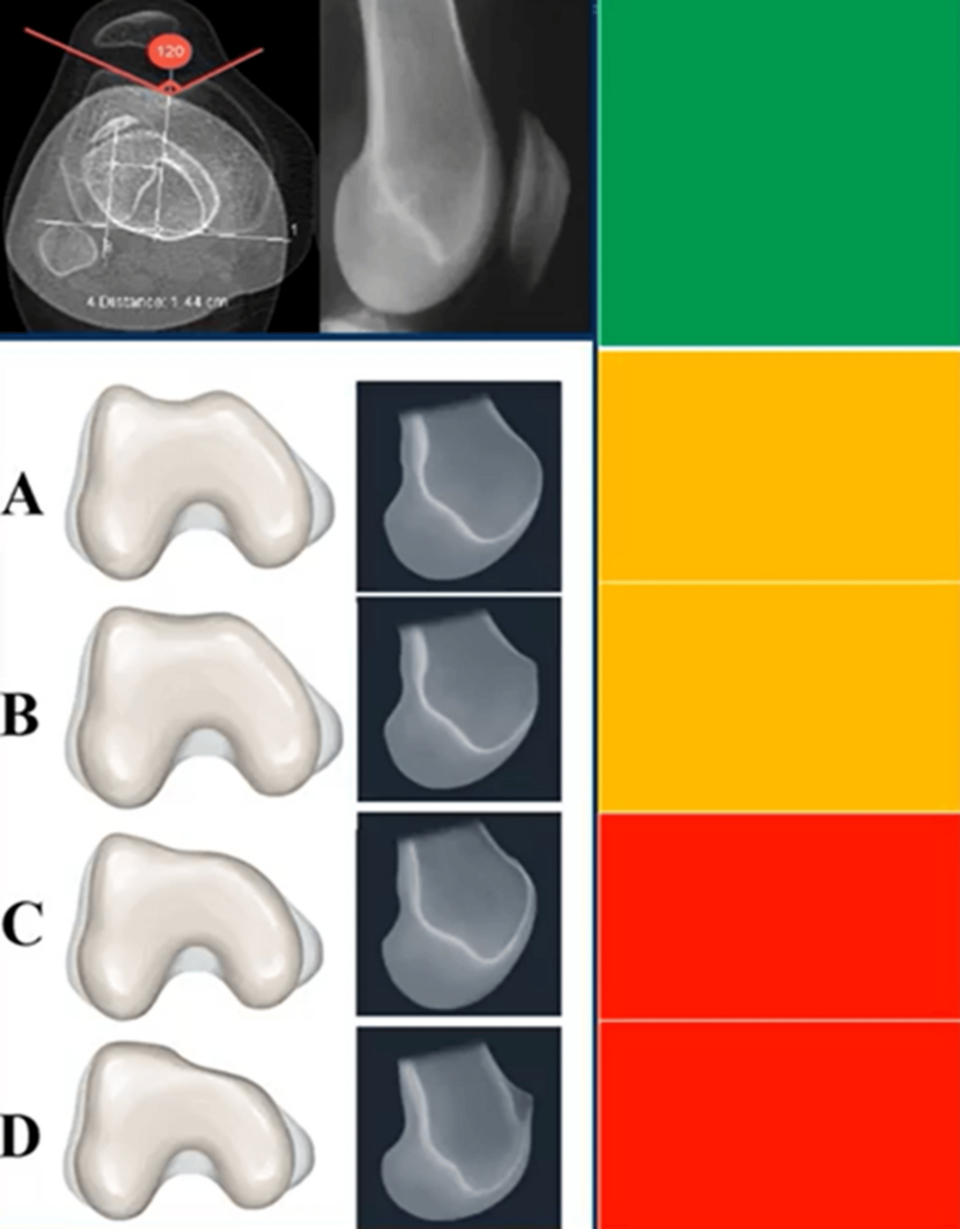
Trochlear dysplasia grading according to the matrix approach. A trochlea exhibiting a sulcus angle of less than 144° is categorized as green, indicating normalcy. De Jour's type A and type B are designated as orange, whereas types C and D are labeled as red. Figures are provided by the authors.

Within the matrix approach, a trochlea exhibiting a sulcus angle of less than 144° is categorized as green, indicating normalcy. De Jour's type A and type B are designated as orange, whereas types C and D are labelled as red (Figure 6).

Patellar dysplasia may manifest as primary, as seen in conditions, such as nail-patella syndrome, or secondary to factors, such as trochlear dysplasia, chronic dislocation, or trauma.

Patella Alta

**Figure 7:**
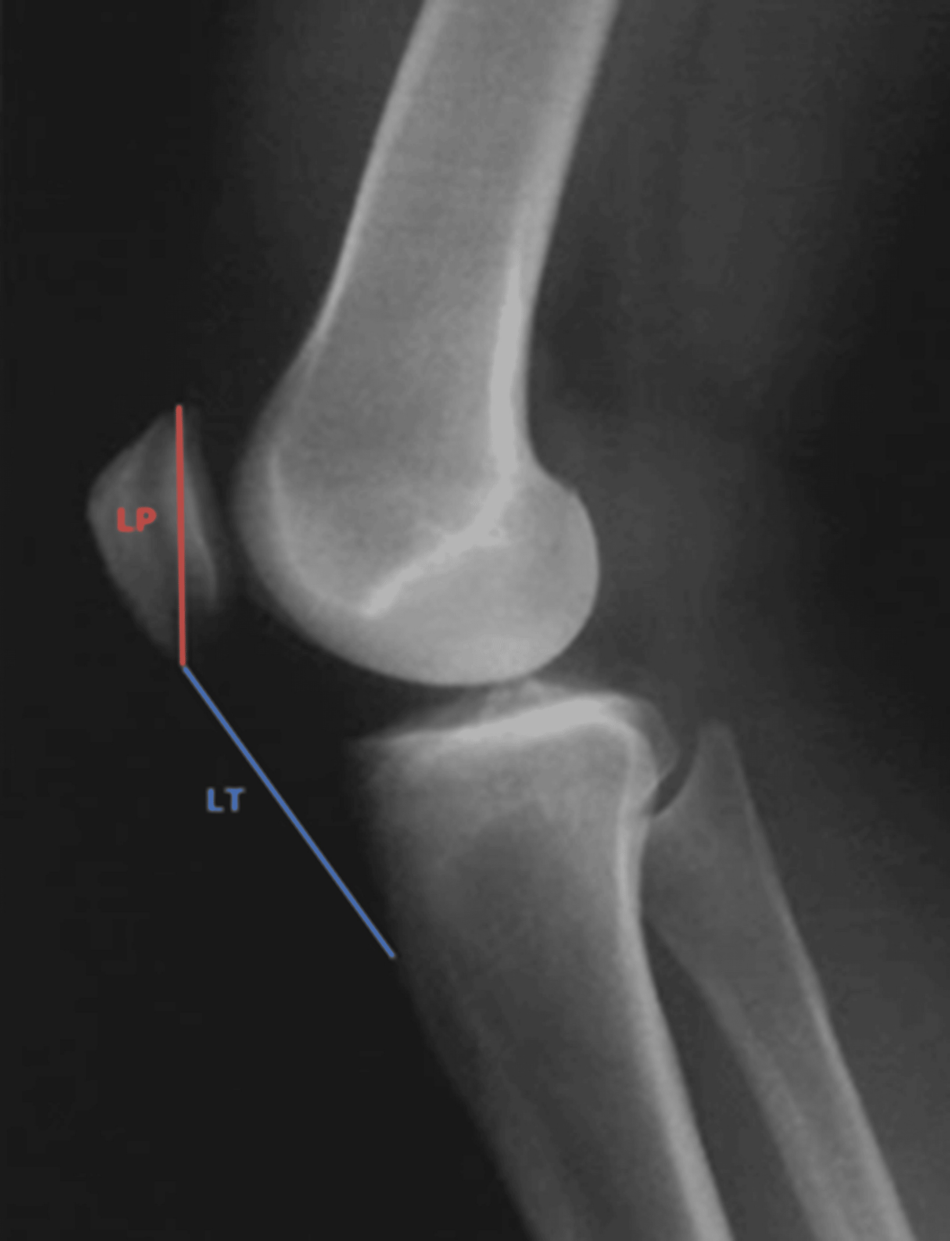
The Insall-Salvati index. Patella alta is characterized by the abnormal and elevated placement of the patella relative to the femur. It is diagnosed when the Insall-Salvati index exceeds 1. Figures are provided by the authors.

Patella alta, characterized by the abnormal and elevated placement of the patella relative to the femur, stands as another significant risk factor associated with patellar instability [9-11]. Precision in measuring patella alta poses challenges, primarily because of the cartilaginous composition of the patella and the tibial tubercle, especially in younger individuals. Although commonly employed, the Insall-Salvati index, which depends on these structures, has inherent limitations. The Insall-Salvati index (Figure 7) compares the length of the patellar tendon (LT) to the length of the patella (LP), typically yielding a value of 1. A value of 1.2 signifies patella alta, while 0.8 indicates patella baja. It is categorized as green when the index falls between 0.8 and 1.2, orange when it ranges from 1.2 to 1.6, and red when it exceeds 1.6.

Tuberosity to Trochlear Groove Distance

**Figure 8:**
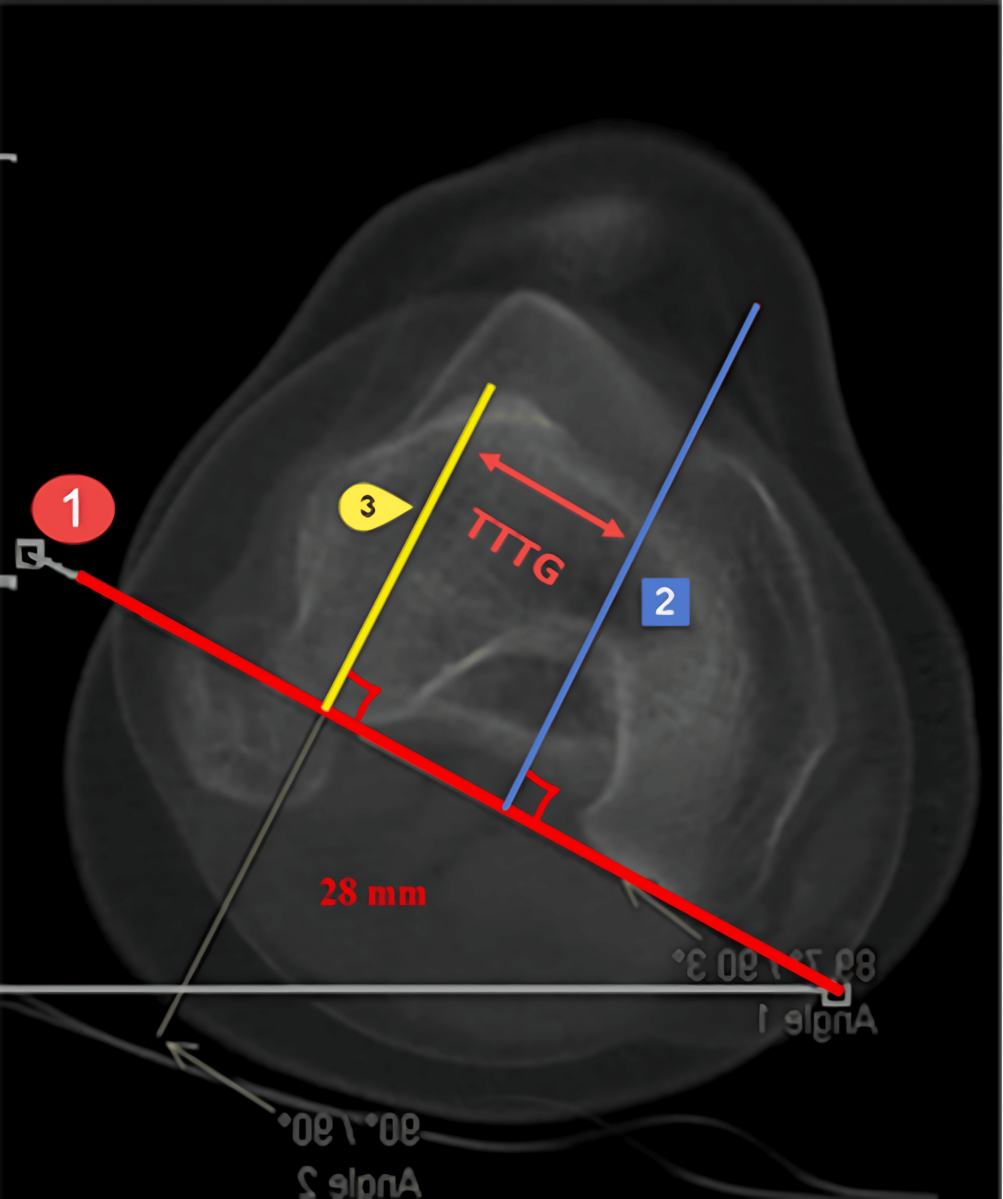
TTTG distance. The TTTG distance is assessed by drawing a line originating from the posterior femoral condyle (marked with red line number 1) and perpendicular lines drawn from the deepest part of the trochlea (indicated by blue line number 2) and the most prominent part of the tibial tuberosity (denoted by yellow line number 3). A typical TTTG distance falls within the range of 9-15 millimeters. Figures are provided by the authors.

The TTTG distance serves as a standard measurement often conducted on CT scans to assess the risk of patellofemoral instability [12,13]. It represents the measurement between the deepest point of the trochlear groove and the tibial tuberosity. Typically, this is evaluated by drawing a line from the posterior femoral condyle and perpendicular lines from the deepest part of the trochlea and the most prominent section of the tibial tuberosity. A normal TTTG distance ranges from 9 to 15 millimeters. Measurements exceeding 20 millimeters are considered severe (red), while values falling in between are categorized as orange or moderate (Figure 8).

Ligaments

Medial Patellofemoral Ligament (mPFL)

The medial patellofemoral ligament (mPFL) serves as a crucial stabilizer of the patella, effectively preventing lateral dislocations. It is a ligamentous structure extending transversely from the posterior aspect of the medial epicondyle, situated approximately 1 cm distal to the adductor tubercle, to the medial aspect of the patella [14]. The avulsion of the mPFL is frequently observed as a small bone fragment on the medial aspect of the patella. However, MRI scans provide a more precise depiction of mPFL abnormalities [3]. mPFL rupture or avulsion is classified as red, a stretched mPFL is denoted as orange, while a normal mPFL is categorized as green.

Generalized Laxity

**Figure 9:**
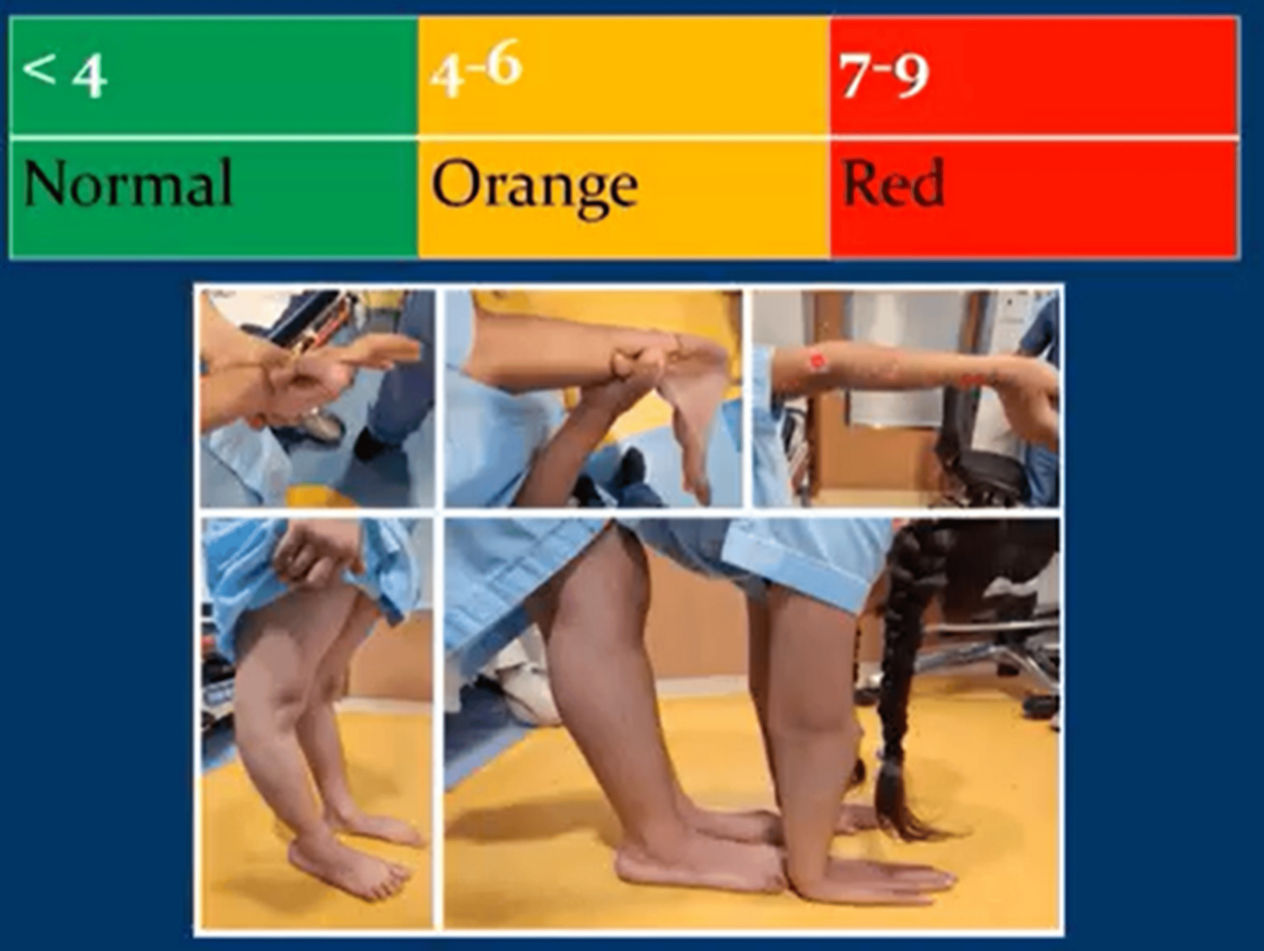
The Beighton score functions as a method for assessing generalized ligament hyperlaxity. A Beighton score of 7 out of 9 or higher is categorized as red, 4-6 out of 9 is classified as orange, and 4 or fewer out of 9 is denoted as green. The above child has a Beighton score of 9 out of 9, categorizing her into the red category of the matrix score. Figures are provided by the authors.

The Beighton score serves as a tool for evaluating generalized ligament hyperlaxity [15]. It comprises a nine-point scale involving the performance of five specific maneuvers (Table 2). A score of 7 out of 9 or higher is categorized as red, 4-6 out of 9 is classified as orange, and 4 or fewer out of 9 is denoted as green (refer to Figure 9).

**Table 2:** The Beighton score. According to the matrix approach, a score of 7 out of 9 or higher is categorized as red, 4-6 out of 9 is classified as orange, and 4 or fewer out of 9 is denoted as green.

The Beighton score
1. Passive dorsiflexion of the fifth metacarpophalangeal joint beyond 90°
2. Passive apposition of the thumb to the flexor aspect of the forearm
3. Passive hyperextension of the elbow beyond 10°
4. Passive hyperextension of the knee beyond 10°
5. Active forward flexion of the trunk with the knees fully extended, allowing the palms of the hands to rest flat on the floor

Muscle

Muscle weakness is assessed using the Medical Research Council (MRC) grading system, which categorizes strength into different grades. Grade 5 signifies normal strength, Grade 4 indicates movement against gravity and resistance, Grade 3 denotes movement against gravity over almost the full range, Grade 2 represents movement of the limb but not against gravity, Grade 1 signifies visible contraction without limb movement, and Grade 0 indicates no visible contraction. In the Matrix approach, Grade 5 is designated with green, Grades 3 and 4 are marked with orange, while Grades 2, 1, and 0 are indicated by red.

Muscle tone can be categorized as normal, low (hypotonia), or increased (hypertonia). Although there are various grading systems for hypertonia and hypotonia separately, in the context of patellar instability, the combined grading published by O’Sullivan et al. is preferred (Table 3) [16]. Grade 0 and +4 are allocated to red, 1+ and 3+ are designated orange, while 2+ is classified as normal.

**Table 3:** Gradings of muscle tone. The combined gradings of hypotonia and hypertonia are preferred in the matrix approach. Grades 0 and +4 are allocated to red, 1+ and 3+ are designated orange, while 2+ is classified as normal. Source: Ref [16]

Gradings of muscle tone
0 indicates no response (flaccidity)
1+ denotes decreased response (hypotonia)
2+ signifies a normal response
3+ indicates an exaggerated response (mild to moderate hypertonia)
4+ represents a sustained response (severe hypertonia)
0 indicates no response (flaccidity)

Trauma

The final two criteria in the Matrix approach pertain to traumatic events and the frequency of dislocations. If patellar dislocation or instability is attributable to a definite traumatic event, it is classified as red. Conversely, if there is no history of trauma, it is categorized as green. Orange is assigned when there is a potentially traumatic event, but uncertainty remains. The habitual occurrence of patellar dislocations refers to their frequency, with red indicating daily dislocations or subluxations, orange indicating monthly occurrences, and green indicating less frequent than once a year.

## Discussion

Patellofemoral instability is a common health problem affecting approximately 43 per 100,000 [1]. A significant portion of these individuals experience recurrent patellar dislocations, anterior knee pain, or premature patellofemoral osteoarthritis, which remains a challenging form of arthritis to manage. Despite the advancements in modern artificial patellofemoral replacement, the outcomes remain suboptimal [17].

Nonoperative treatments often prove ineffective, with reported recurrence rates nearing 50% [18,19]. This has spurred the development of numerous surgical interventions in pursuit of improved outcomes. However, the results of these early interventions have not been universally successful. Hence, there exists a plethora of over 100 surgical interventions recommended for patellofemoral instability, none of which have achieved universal success. Consequently, surgeons may hesitate to recommend surgery, leaving patients inadequately treated.

Our understanding of patellofemoral instability has evolved significantly over the past decade, leading to the development of the matrix approach. This evolution has been driven by critical analysis of the literature, thorough assessment of patients, and careful evaluation of treatment outcomes. We have gleaned valuable insights from both our successes and failures in managing this condition. The current iteration of the Matrix approach was implemented into our practice in 2020, yielding excellent outcomes.

While several classifications and approaches for patellofemoral instability exist, they often fall short of adequately addressing important risk factors or grading them in a manner that guides treatment effectively. For instance, Hinton et al. proposed categorizing patients with patellar instability into two groups: LAACS and TONES (refer to Table 2) [20].

**Table 4:** The LAACS and TONES classification. TONES patients typically exhibit athleticism, experience poor tolerance to instability, and are at a higher risk of developing recurrent instability without surgical intervention. Conversely, LAACS patients tend to have less traumatic instability, leading to fewer osteochondral fractures and less soft tissue disruption. Their instability also minimally disrupts daily routines, making them more amenable to nonoperative management and activity modification. Source: Ref [20]

LAACS	TONES
L: Laxity, generalized and lower-aged at initial dislocation	T: Traumatic, sports-related mechanism
A: Atraumatic in nature	O: Older at initial dislocation, osteochondral fracture more common
A: Abnormal patellofemoral architecture	N: Normal patellofemoral architecture, normal alignment
C: Chronic in nature, contralateral involvement	E: Equal sex distribution
S: Sex dependent with greater number of females	S: Single occurrence, single leg involvement

According to Hinton's classification, TONES patients (Table 2) typically exhibit athleticism, experience poor tolerance to instability, and are at a higher risk of developing recurrent instability without surgical intervention. Conversely, LAACS patients (Table 2) tend to have less traumatic instability, leading to fewer osteochondral fractures and less soft tissue disruption. Their instability also minimally disrupts daily routines, making them more amenable to nonoperative management and activity modification.

Our concern with approaches like Hinton's and similar ones is that they attempt to fit patients into predefined classifications rather than identifying the underlying causes of patellar instability, grading their severity, and addressing them accordingly. These approaches tend to underestimate the etiological significance of femoral and tibial rotational deformities and oversimplify patellofemoral abnormalities as a single entity, despite the potential presence of multiple pathologies, including patella alta, trochlear dysplasia, patellar dysplasia, and high TTTG distance.

In contrast, the matrix approach adopts an individualized patient-based classification, recognizing that each patient is unique in terms of predisposing factors and their severity. To help educate healthcare professionals on applying the matrix approach to individual patients, we have produced an educational video [Video 1], which is freely available for viewing.

**Video 1:** The matrix approach to patellar instability/dislocation. An overview of the matrix approach with examples of its application in clinical scenarios. The video is provided by the authors.

## Conclusions

The matrix approach for patellofemoral instability offers a comprehensive framework for understanding the etiopathology of instability in individual patients. It utilizes a grading system akin to traffic lights, facilitating easy comprehension and reproducibility. This approach informs decision-making by categorizing predisposing factors as green, orange, or red, thereby guiding the selection of interventions, whether singular or combined, to address instability effectively. Factors graded as orange present ongoing challenges and are the focus of continuous research aimed at identifying optimal management strategies.
